# Prognostic significance of K-ras mutations in pancreatic cancer: a meta-analysis

**DOI:** 10.1186/s12957-016-0888-3

**Published:** 2016-05-16

**Authors:** Lian-yuan Tao, Ling-fu Zhang, Dian-rong Xiu, Chun-hui Yuan, Zhao-lai Ma, Bin Jiang

**Affiliations:** Department of General Surgery, Peking University Third Hospital, No. 49, Hua Yuan North Rd, Hai Dian District, Beijing, 100191 China

**Keywords:** K-ras, Pancreatic cancer, Prognosis, Meta-analysis

## Abstract

**Background:**

K-ras gene mutations are common in patients with pancreatic cancer (PC); however, their prognostic value for PC remains inconclusive. This meta-analysis was performed to quantitatively evaluate the association between K-ras mutations and survival in patients with pancreatic cancer.

**Methods:**

We performed a comprehensive search of electronic sources including MEDLINE (via PubMed), Web of Science, and the Cochrane Library. The search covered a publication period from inception to November 2015.

**Results:**

Seventeen studies with a total of 2249 patients with pancreatic cancer were included in the tissue detection of this study. The meta-analysis indicated a significant association between mutant K-ras genes and overall survival (OS) (HR = 1.51, 95 % CI 1.32–1.72, *P* < 0.001). Moreover, further subgroup analyses by ethnicity, publication year, therapy method, cancer resectability, and gene detection method all revealed that pancreatic cancer patients with the K-ras mutation had significantly poorer OS (*P* < 0.05). And results from four studies with 225 patients focused on plasma K-ras mutations enhanced such association (HR = 2.23, 95 % CI 1.69–2.95, *P* < 0.001).

**Conclusions:**

As a prediction of poor prognosis, the detection of K-ras mutations may be a useful prognostic factor for pancreatic cancer patients.

## Core tip

To our knowledge, this is the first meta-analysis of all eligible studies on the prognostic role of the K-ras mutation in patients with pancreatic cancer. In populations of both Caucasian and Asian descent, patients with pancreatic cancer harboring K-ras mutations tend to get a worse survival. K-ras mutations may represent a useful prognostic factor to stratify patients with high risk and develop specific treatments for these patients in clinical applications.

## Background

Pancreatic cancer (PC) has a 5-year survival rate of less than 5 %, is one of the most aggressive malignancies, and represents a leading cause of cancer-related mortality [1, 2]. Most patients were diagnosed when they got jaundice, and imaging examination was the most effective diagnosis tool; however, those patients may get an advanced stage and even lost the opportunity for operation, as operation is still the only effective treatment for PC. What is more, even patients received a curative operation, the prognosis is still unsatisfactory.

As a member of the Ras gene family, K-ras plays a key role in Ras/mitogen-activated protein kinase signaling. Somatic mutation in K-ras mutations have been shown to be early events in the carcinogenesis of human pancreatic cancer [3, 4]. Approximately 80 % of K-ras mutations in pancreatic cancer involve codon 12; others are located in codons 13, 61, and 1 [5–7]. K-ras mutations have been demonstrated to enhance cellular proliferation and induce malignant transformation, and their continuous activation played a key role in the development and maintenance of pancreatic cancer [8].

Recent meta-analyses have suggested that K-ras mutations can be used as useful biomarkers for the early detection of pancreatic cancer [9, 10]. It has been reported positive in about 65 % patients with PC. Although it expressed in most pancreatic cancer patients, a sensitivity of 65 %, sometimes even lower to 36 %, limits its diagnosis application [9, 10]. However, its application in the predication of prognosis and guidance of treatment may be much more valuable. Although many recent studies evaluated K-ras gene mutations that appeared to influence the prognosis and patterns of gene expression [11, 12], the use of K-ras mutations as a prognostic factor for pancreatic cancer remains inconclusive. To clarify the role of K-ras mutations in the prognosis of pancreatic cancer, we performed the present comprehensive meta-analysis. The detection sources could be tissues or plasma, we tend to explore both of the sources respectively. To our knowledge, this study was the first meta-analysis of all eligible studies on the prognostic role of the K-ras mutation in patients with pancreatic cancer.

## Methods

### Literature search

A systematic literature search was carried out in MEDLINE (via PubMed), Web of Science, and the Cochrane Library to screen for cohort/case-control studies characterizing the association between K-ras mutation and prognosis in PC patient. The search terms were pancreatic or pancreas neoplasms, ras or K-ras gene, survival, and prognosis, which covered the publication period from inception to November 2015. The meta-analysis was performed using the STATA statistical software. Review articles were also screened to search for relevant original studies. Only articles published in English were included in our meta-analysis.

### Study selection criteria

Studies deal with the comparison between PC patient with and without K-ras mutation fulfilling the following criteria were considered to satisfy the inclusion criteria of present study: (1) cohort studies, nested case-control studies, or case-control studies focusing on the prognostic value of K-ras mutant type in patients with pancreatic cancer; (2) gene amplification status of K-ras was detected in surgical or plasma specimens; (3) all patient diagnoses of pancreatic cancer were confirmed through histopathologic detection; (4) sufficient data were provided to calculate hazard ratios (HR) for OS comparing mutant K-ras with wild-type K-ras patients; and (5) more than ten patient samples with K-ras mutation were included in the original studies because small sample size may be vulnerable to selection bias. If more than one study by the same authors (using the same case series) was published, the study with the largest sample size was included. The data collected from surgical tissues and plasma specimens were divided in two different groups and analyzed, respectively.

### Data extraction and methodological quality assessment

Two reviewers (LY Tao and LF Zhang) firstly screened the titles and/or abstracts of all articles independently; we resolved cases with any disagreements through discussion and careful reexaminations. The following variables from studies were extracted with a pre-designed spreadsheet: first author, year of publication, source of publication, country, patients’ ethnicity, study design, total number of cases, detection method of K-ras expression, mutated sites, treatment method, and OS. Quality assessment of the included studies was conducted based on the Newcastle-Ottawa Scale (NOS) criteria (targeting the quality of non-randomized studies) [13]. The NOS criteria apply a “star” rating system ranges from 0 (worst) to 8 (best) for the judgment of methodological quality, which was based on selection, comparability, and outcome. We set 5 star as the cutoff value of our analysis, as article with NOS ≥5 was qualified enough for a meta-analysis. Conflicting evaluations or inconsistent data from the eligible studies were resolved through discussion or by asking a verdict by a third arbitrator (DR Xiu).

### Statistical analysis

The effects of K-ras gene mutations on OS were assessed using the overall HR and 95 % confidence interval (95 % CI). Data from Kaplan-Meier survival curves were collected through Engauge Digitizer version 4.1 (free software downloaded from http://sourceforge.net) when the HR was not provided, and the minimum and maximum follow-up periods were obtained from the articles. Heterogeneity between studies was estimated using both the Cochran’s Q statistic (which considered significant at *P* < 0.10 [14]). A fixed effects model (the Mantel-Haenszel method) was used for the calculation of Crude HRs when there was no statistically significant heterogeneity (*Q* test with *P* > 0.10). Otherwise, the random effects model (the DerSimonian Laird method) was conducted. The significance of the pooled estimate was determined using the *Z* test.

Subgroup analyses were performed based on ethnicity, publication years, detection methods, tumor resectability, and treatment methods. Besides, a sensitivity analysis was performed using the sequential omission of individual studies to assess the quality and consistency of the results. Begg’s funnel plots were also constructed to evaluate the effect of publication bias on this study, and Egger’s linear regression test was further performed to evaluate the symmetry of these funnel plots [15]. All meta-analyses were calculated using Stata software 12.0 (Stata Corp LP, College Station, TX, USA). All tests were two-sided with *P* < 0.05 as statistically significant.

## Results

### Description and quality assessment of studies

**Fig. 1:**
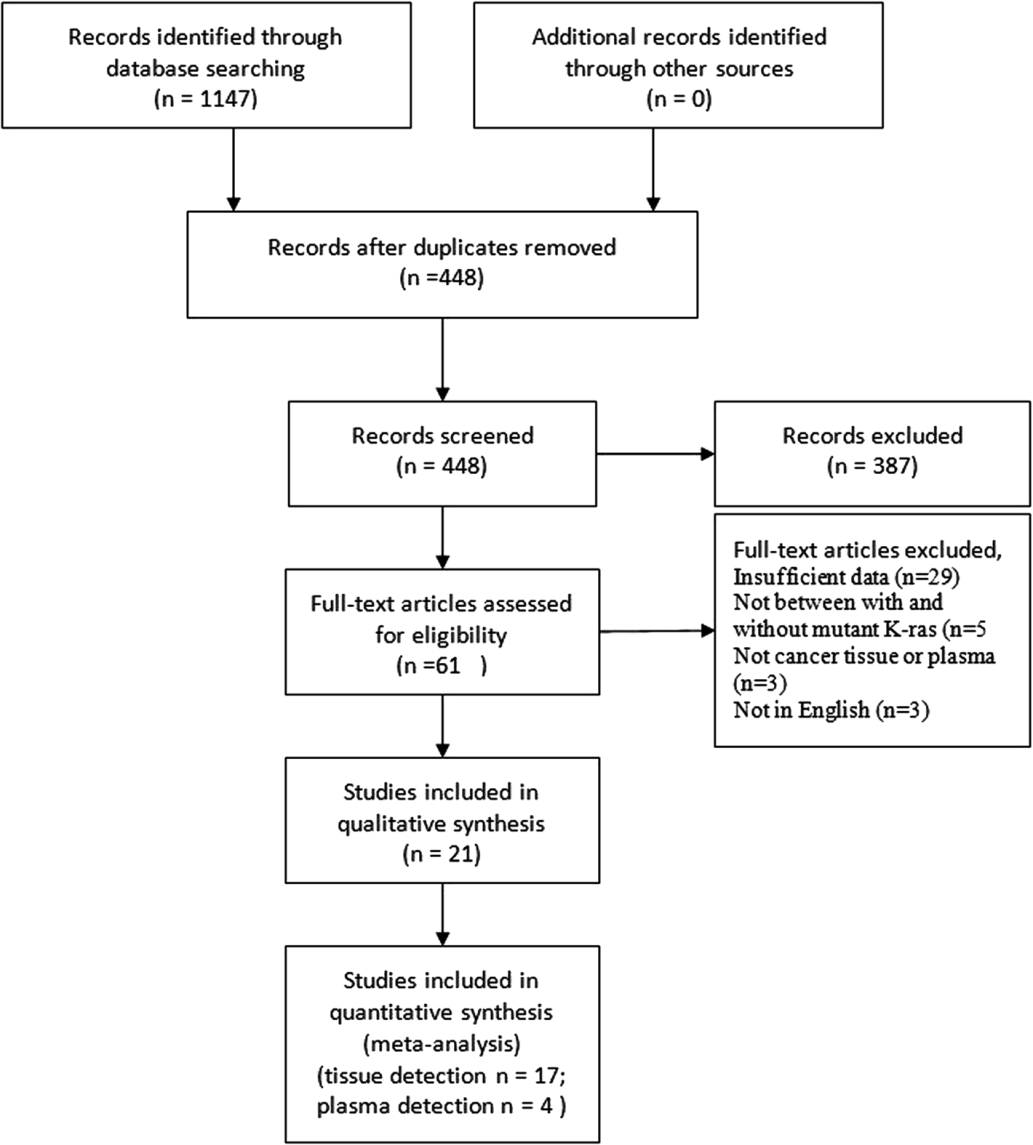
Flowchart of the search history in this meta-analysis

A total of 1147 studies meeting the search strategy were initially identified, and 699 duplicates were excluded, leaving 448 articles. After a review of their titles and abstracts, 387 articles were excluded. Another 61 articles were excluded after full text identification, leaving 17 studies for tissues detection and 4 studies for plasma detection that met our criteria for this meta-analysis (Fig. 1). As for tissue detection, a total of 2249 (1302 males and 947 females) pancreatic cancer patients, including 1261 patients in the K-ras mutant group and 988 patients in the wild-type group, were involved in present meta-analysis. A summary of the characteristics and methodological quality of the included studies are shown in Table 1. Four studies with a total of 225 patients were included in the analysis of plasma detection, which are listed in Table 2.

**Table 1:** Characteristic summary of studies included in the meta-analysis

The author	Year	Country	Ethnicity	Age (years)	Gender (M/F)	Treatment	Detection method	Outcome	NOS
Allison, D.C. [16]	1998	USA	Caucasians	63 (32–80)	40/36	Operation	Enriched PCR and oligonucleotide hybridization	OS	6
Boeck, S [17]	2013	Germany	Caucasians	64 (32–78)	105/68	Chemotherapy	Pyrosequencing	OS	7
Kim, S.T. [18]	2011	Korea	Asians	≥60, *n* = 84 < 60, *n* = 52	99/37	Chemotherapy	DS	OS	7
Ogur, T [19]	2014	Japan	Asians	65 (35–84)	146/96	Chemotherapy	RT-PCR	OS	6
Schultz, N.A. [6]	2012	Denmark	Caucasians	63 (33–85)	88/82	Operation	DS	OS	7
Shin, S.H. [20]	2013	Korea	Asians	60 (22–78)	139/95	Operation	PCR-RFLP	OS	8
Sinn, B.V. [7]	2014	Germany	Caucasians	≥65, *n* = 81; <65, *n* = 72	84/69	Operation	DS	OS	7
Franko, J [21]	2008	USA	Caucasians	68 ± 12	26/24	Operation and chemotherapy	DS	OS	6
Da Cunha Santos, G [22]	2010	Canada	Caucasians	62 (40–85)	64/53	Chemotherapy	PCR and BS	OS	7
Fensterer, H [5]	2013	Germany	Caucasians	62.7	36/30	Operation and chemotherapy	High-resolution melting assay	OS	6
lkeda, N [23]	2001	Japan	Asians	63.7 (47–80)	37/11	Operation and chemotherapy	DS	OS	7
Kwon, M.J. [24]	2011	Korea	Asians	63 (45–86)	37/35	Operation and chemotherapy	RT-PCR	OS	8
Lee, J [25]	2007	Korea	Asians	≥60, *n* = 47; <60, *n* = 19	51/15	Chemotherapy	DS	OS	5
Oh, D.Y. [26]	2012	Korea	Asians	57.3 (39–77)	24/16	Chemotherapy	DS	OS	6
Salek, C [27]	2009	Czech	Caucasians	63 ± 10.5 (40–84)	28/25	Chemotherapy	GenoScan	OS	6
Kinugasa, H [28]	2015	Japan	Asians	66 (47–85)	54/21	NR	Digital PCR	OS	7
Talar-Wojnarowska, R [29]	2005	Poland	Caucasians	47–76	10/16	Operation	PCR-RFLP	OS	6

*DS* direct sequencing, *BS* bidirectional sequencing, *NR* not reported, *RT*-*PCR* reverse transcription polymerase chain reaction, *PCR-RFLP* polymerase chain reaction-restriction fragment length polymorphism, *NOS* Newcastle-Ottawa Scale (NOS) criteria (targeting the quality of non-randomized studies)

**Table 2:** Characteristic summary of studies that detected the K-ras gene in plasma

Author	Year	Country	Ethnicity	Age (years)	Gender (M/F)	K-ras (mutant/wild)	HR (95 % CI)	Outcome	Sites
Kinugasa, H [28]	2015	Japan	Asians	66 (47–85)	54/21	47/28	1.84 (1.1–3.25)	OS	12, 13, 61
Castells, A [12]	1999	Spain	Caucasian	NR	NR	12/32	1.51 (1.02–2.23)	OS	12
Chen, H.H. [30]	2010	China	Asians	60 (37–78)	57/34	30/61	7.39 (3.7–14.9)	OS	12
Yadama, T [31]	1998	Japan	Asians	63.9 (35–78)	11/4	11/4	4.7 (2.8–21.2)	OS	12
Combined							2.23 (1.69–2.95)	OS	

*NR* not reported, *Sites* sites of K-ras mutations involved, such as codons 12, 13, and 61

### Quantitative data synthesis

**Fig. 2:**
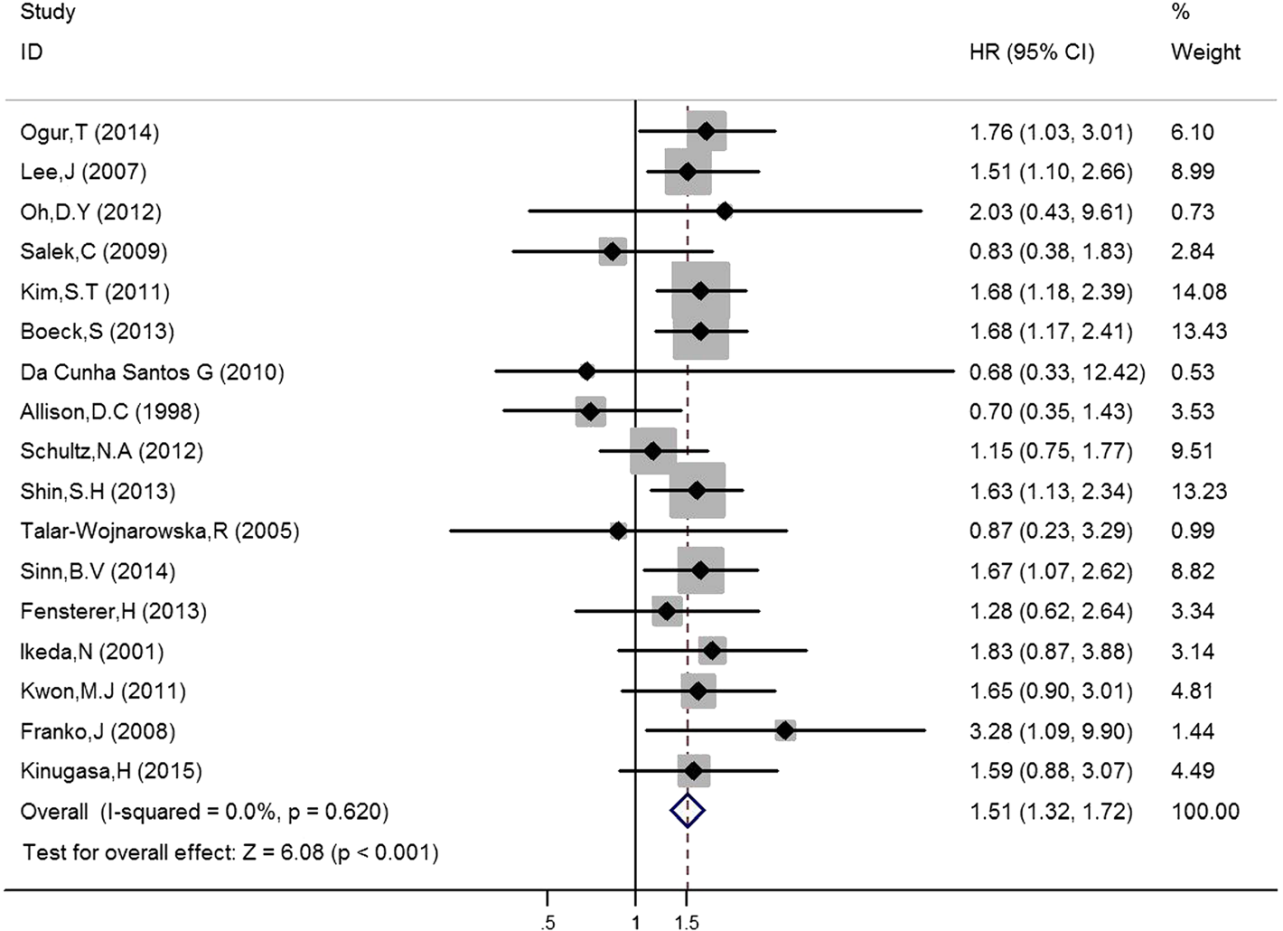
Forest plots for the relationships between K-ras gene mutations and prognosis in patients with pancreatic cancer

The meta-analysis results suggested that K-ras gene mutations were significantly associated with poorer OS (HR = 1.51, 95 % CI 1.32–1.72, *P* < 0.001; *P* for heterogeneity 0.62, fixed effects model) (Fig. 2). HRs for OS comparing the K-ras mutant type group with the wild-type group is summarized in Table 3.

**Table 3:** The hazard ratios for overall survival comparing K-ras mutations with the wild-type in the included studies

The author	Year	K-ras (mutant/wild)	HR (95 % CI)	*P* value	Sites
Allison, D.C. [16]	1998	64/12	0.70 (0.35–1.43)	0.330	NR
Boeck, S [17]	2013	121/52	1.68 (1.17–2.41)	0.005	12, 13
Kim, S.T [18]	2011	71/65	1.68 (1.18–2.39)	0.001	12, 13
Ogur, T [19]	2014	214/28	1.76 (1.03–3.01)	0.040	12
Schultz, N.A. [6]	2012	136/34	1.15 (0.75–1.77)	0.510	12, 13, 61
Shin, S.H. [20]	2013	126/108	1.63 (1.13–2.34)	0.001	12, 13
Sinn, B.V. [7]	2014	105/48	1.68 (1.07–2.62)	0.023	12, 13, 61
Franko, J [21]	2008	31/19	3.28 (1.09–9.90)	0.035	12, 13, 1
da Cunha Santos, G [22]	2010	92/25	0.68 (0.33–12.42)	0.300	12, 13
Fensterer, H [5]	2013	45/21	1.28 (0.62–2.64)	0.180	12, 13
lkeda, N [23]	2001	33/15	1.83 (0.87–3.88)	0.317	12
Kwon, M.J. [24]	2011	34/38	1.65 (0.90–3.01)	0.159	12, 13, 1
Lee, J [25]	2007	33/33	1.51 (1.01–2.66)	0.030	12
Oh, D.Y. [26]	2012	19/21	2.03 (0.43–9.61)	0.158	12, 13
Salek, C [27]	2009	36/17	0.83 (0.38–1.83)	0.636	12, 13
Kinugasa, H [28]	2015	47/28	1.59 (0.88–3.07)	0.124	12, 13, 61
Talar-Wojnarowska, R [29]	2005	20/6	0.87 (0.23–3.29)	0.580	12

*NR* not reported, *Sites* sites of K-ras mutations involved, such as codons 12, 13, and 61

Results from a subgroup analysis by ethnicity indicated that K-ras mutant patients had poorer OS among both Caucasia and *n* Asian populations (HR = 1.35, 95 % CI 1.10–1.64, *P* = 0.000 % and HR = 1.65, 95 % CI 1.38–1.97, *P* < 0.001, respectively; both *P* for heterogeneity >0.1, fixed effects models). Because the publication number of K-ras-related articles focus on pancreatic cancer was elevated significantly after 2010, we chose the year of 2010 as a cutoff point. The further stratified analyses by publication year suggested that, despite no significant association between K-ras mutation and OS prior to 2010, which included the year of 2010 (HR = 1.27, 95 % CI 0.96–1.69, *P* = 0.098; *P* for heterogeneity >0.1, fixed effects model), the K-ras mutant patients had worse OS than the patients without K-ras mutations after 2010 (HR = 1.58, 95 % CI 1.36–1.83, *P* < 0.001; *P* for heterogeneity >0.1, fixed effects model). With the exception of the study by Kinugasa H [28], which failed to provide detailed information about the treatment, subgroup analyses by the respectability (those who underwent an operation) of cancer indicated that K-ras mutant patients had worse OS whether the cancers were resectable or not (HR = 1.44, 95 % CI 1.19–1.74, *P* < 0.001 and HR = 1.57, 95 % CI 1.30–1.91, *P* < 0.001, respectively; both *P* for heterogeneity >0.1, fixed effects models). Additionally, the subgroup analysis according to treatment methods revealed a statistically significant difference in OS between the K-ras gene mutant group and the wild-type group (operation only: HR = 1.35, 95 % CI 1.09–1.69, *P* = 0.005 %; chemotherapy only: HR = 1.57, 95 % CI 1.30–1.91, *P* < 0.001; operation and chemotherapy: HR = 1.71, 95 % CI 1.18–2.48, *P* = 0.005, respectively; All *P* for heterogeneity >0.1, fixed effects models). Finally, a subgroup analysis according to gene detection methods also showed that the K-ras gene mutation was significantly associated with poorer OS (HR = 1.46, 95 % CI 1.22–1.75, *P* < 0.001 and HR = 1.57, 95 % CI 1.29–1.90, *P* = 0.005, respectively; *P* for heterogeneity >0.1, *both* fixed effects models) (Table 4).

**Table 4:** Subgroup analysis of the association between K-ras mutations and overall survival of patients with pancreatic cancer

Subgroup	No. of patients with mutant K-ras	No. of patients without mutant K-ras	HR (95 % CI)	Heterogeneity *I* ^2^ (%)	Heterogeneity *P* value
Ethnicity					
Caucasian	650	234	1.35 (1.10–1.63)	28.0	0.195
Asian	586	327	1.65 (1.32–1.72)	0.0	0.136
Publication year					
Before 2010	309	127	1.27 (0.96–1.69)	33.1	0.175
After 2010	927	434	1.58 (1.36–1.83)	0.0	0.963
Tumor resectability					
Resectable	594	301	1.44 (1.19–1.74)	14.3	0.315
Unresectable	586	241	1.57 (1.30–1.91)	0.0	0.686
Treatment					
Operation	451	208	1.35 (1.09–1.69)	35.7	0.183
Chemotherapy	586	241	1.57 (1.30–1.91)	0.0	0.686
Operation + chemotherapy	143	93	1.71 (1.18–2.48)	0.0	0.574
Detection methods					
Sequencing	428	235	1.57 (1.29–1.90)	0.0	0.644
Other methods	808	326	1.46 (1.22–1.75)	2.0	0.421

To further evaluate the prognostic value of K-ras mutations in pancreatic cancer, we listed characteristics from the four studies that focused on the relationship between plasma K-ras mutations and pancreatic cancer prognosis [12, 28, 30, 31]. K-ras mutations in all four studies revealed a significant association with poorer OS, and the combined HR also indicated a strong association (HR = 2.23, 95 % CI 1.69–2.95, *P* < *0.001*; *P* for heterogeneity <0.1, random effects model), (Table 2).

### Evaluation of heterogeneity and publication bias

**Fig. 3:**
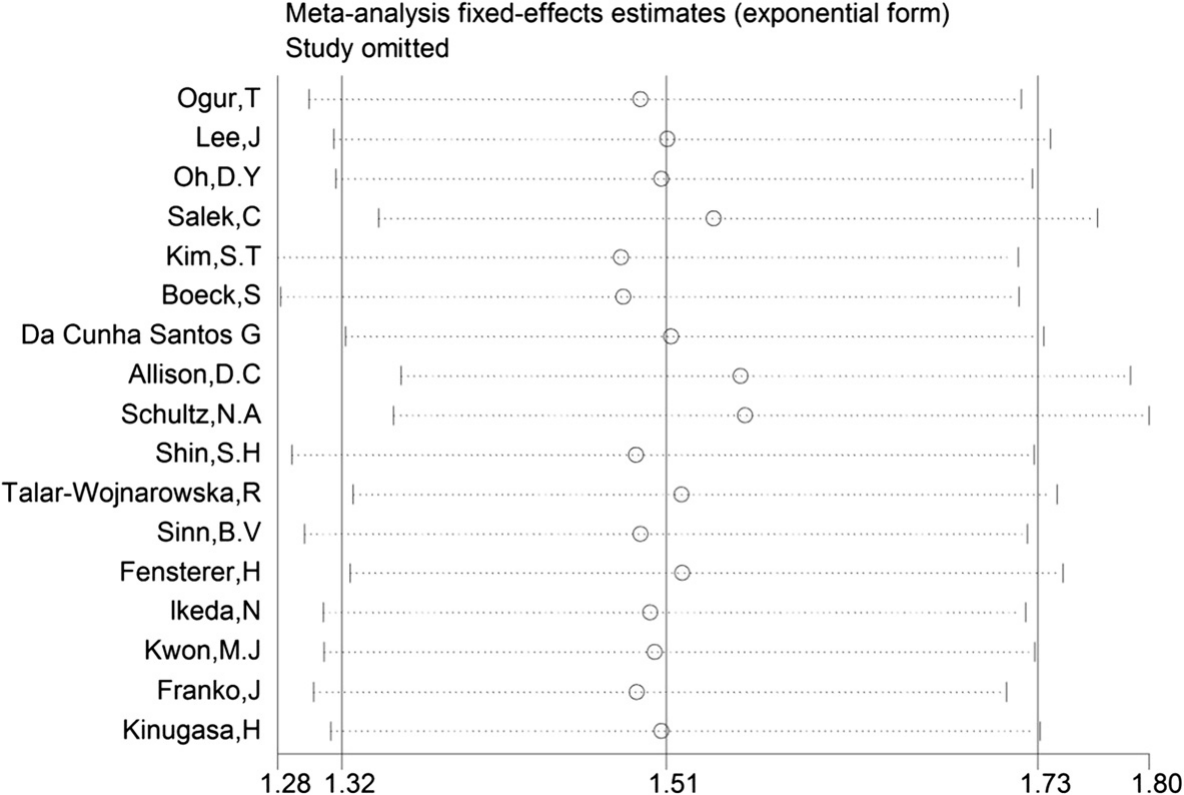
Sensitivity analysis for the pooled HRs of the differences in OS between K-ras gene mutations and wild-type pancreatic patients

**Fig. 4:**
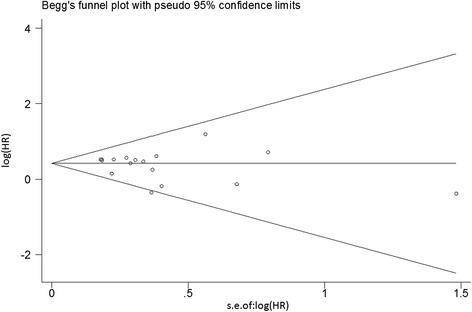
Begg’s funnel plots of the prognostic role of K-ras gene mutations in pancreatic cancer patients. *Each point* represents a separate study for the indicated association. Log(HR) natural logarithm of HR; *horizontal line* means the magnitude of the effect. Note: A funnel plot with ~95 % confidence limit was used (Egger’s test: *t* = −0.95, *P* = 0.356)

The results of the sensitivity analysis suggested that no individual studies significantly affected the pooled HRs (Fig. 3). The shapes of Begg’s funnel plots and the result of Egger’s linear regression test (*P* = 0.356) did not reveal evidence of obvious publication bias (Fig. 4).

## Discussion

In our work, the combined HR for 17 studies evaluating the correlation between K-ras mutations and overall survival of patients with pancreatic cancer was 1.51 (95 % CI 1.32–1.72, *P* = 0.000), which indicated that K-ras mutations have negative prognostic value in pancreatic cancer. In our subgroup analyses, the HR for both Caucasian and Asian populations implied that pancreatic cancer patients harboring K-ras mutations also tend to get a worse survival. Furthermore, subgroup analyses according to tumor resectability and the treatment and detection methods of K-ras still revealed that K-ras gene mutations were strongly correlated with poorer prognoses in patients with pancreatic cancer. Notably, the K-ras mutation was only significantly correlated with poorer OS after 2010 (not included 2010). However, the combined HR of K-ras mutation before is 1.27, which still supports our conclusion. The reason why the insignificant association may be the small sample sizes of the studies.

Detection of K-ras mutations in circulating DNA, which can be performed before and after operation, is much more convenient than the application of tissue samples. The occurrence or absence of K-ras mutations in the peripheral blood might reflect different tumor stages [32]. The detection of K-ras mutations in the peripheral blood could reflect the tumor burden of individual PC patients, and in turn predict a prognosis. During the analysis of plasma samples, all the four studies have a significant association between K-ras mutations and poorer OS, and a higher combined HR of 2.23 further agree with the prognostic value of mutant K-ras. All the data above demonstrated the feasibility of K-ras mutations as a predictor of prognosis of PC patients. It is of great significance to one of the most malignant tumor, which has a 5-year survival rate of less than 5 %. For patients with expression of K-ras mutations, more frequently postoperative re-examination and follow-up survey may be needed and more proactive therapeutic schedule of postoperative adjuvant therapy may be necessary when compared to the negative expressed patients.

Other than a useful prognosis predictor of pancreatic cancer, the detection of mutant K-ras may make it possible to develop new therapeutic approaches. As a member of the Ras gene family, K-ras plays a key role in Ras/mitogen-activated protein kinase signaling. Somatic mutation in K-ras mutations have been shown to be early events in the carcinogenesis of human pancreatic cancer [3, 4]. To blockade the Ras signaling pathway, it has been proposed that cancer vaccines that stimulate immunity against mutant Ras proteins and antisense therapy that blocks the translation of mutant Ras gene could be applied in the treatment after operation [33]. Evidence has indicated that K-ras expression and the growth and invasiveness of PC cell lines can be inhibit by K-ras antisense oligodeoxynucleotide (K-ras-ASODN). Similar effects also can be identified in the models of PC by intraperitoneal injection of adenovirus [34].

This study has several limitations. First, current samples of available studies were relatively small with 17 for tissue detection and only 4 for plasma detection. However, such number is enough for a meta-analysis, and we even give a subgroup analysis of tissue detection studies. Second, the definition of resectability (between centers and surgeons) and the treatment after operation like chemotherapy may be different between the studies. Such limitation is hard to control for the treatment of pancreatic cancer is still under controversy. Lastly, data about tumor stage or sample size were not provided in most included articles, and the divide of stage for analysis was also not unified, which prevent a further subgroup analysis. Despite those shortcomings, the effect of K-ras on survival was consistent in nearly all of the included studies, and no studies reported a favorable outcome in patients with K-ras mutations.

## Conclusions

In conclusion, K-ras gene mutations are associated with a poorer prognosis in patients with pancreatic cancer. It may represent a useful prognostic factor to stratify patients with high risk and in developing specific treatments for these patients in clinical applications.

## Institutional review board statement

This study was approved by the Clinical Ethics Committee of Peking University Third Hospital.
